# Combining FORGE Score and Histopathological Diagnostic Criteria of Atypical Meningioma Enables Risk Stratification of Tumor Progression

**DOI:** 10.3390/diagnostics11112011

**Published:** 2021-10-29

**Authors:** Johannes Wach, Tim Lampmann, Ági Güresir, Hartmut Vatter, Albert J. Becker, Michael Hölzel, Marieta Toma, Erdem Güresir

**Affiliations:** 1 Department of Neurosurgery, University Hospital Bonn, 53127 Bonn, Germany; tim.lampmann@ukbonn.de (T.L.); agi.gueresir@ukbonn.de (Á.G.); hartmut.vatter@ukbonn.de (H.V.); erdem.gueresir@ukbonn.de (E.G.); 2 Department of Neuropathology, University Hospital Bonn, 53127 Bonn, Germany; albert.becker@ukbonn.de; 3 Institute of Experimental Oncology, University Hospital Bonn, 53127 Bonn, Germany; michael.hoelzel@ukbonn.de; 4 Institute of Pathology, University Hospital Bonn, 53127 Bonn, Germany; marieta.toma@ukbonn.de

**Keywords:** atypical meningioma, FORGE, prediction, recurrence

## Abstract

More than 50% of atypical meningiomas regrow within 5 years after surgery. FORGE score is a newly created tool to estimate the MIB-1 index in cranial meningiomas. In this investigation, we aimed to assess the predictive value of the FORGE score in combination with major diagnostic criteria of atypical meningioma (brain invasion, mitotic count ≥ 4) regarding recurrence in atypical meningiomas. We included patients operated on primary atypical meningiomas in our center from 2011 to 2019. The study included 71 patients (58% women, median age 63 years). ROC curves revealed a superiority of FORGE score combined with histopathological diagnostic criteria of atypical meningioma (AT-FORGE) in the prediction of tumor progression compared to FORGE score only (AUC: 0.72; 95% CI: 0.54–0.91, cut-off: ≥5/<5, sensitivity: 75%, specificity: 78%). Patients with an AT-FORGE score ≥ 5 had a shorter time to tumor progression (32.8 vs. 71.4 months, *p* < 0.001) in the univariable analysis. Multivariable cox regression analysis revealed significant predictive value of Simpson grade > II, presence of multiple meningiomas and AT-FORGE score ≥ 5 for tumor progression. The combination of histopathological diagnostic criteria for atypical meningioma with FORGE score might facilitate an effective identification of patients with an atypical meningioma who have an increased risk of tumor progression.

## 1. Introduction

Atypical meningiomas World Health Organization (WHO) grade 2 comprise an intermediate risk group of tumors between benign (WHO grade 1) and anaplastic or malignant (WHO grade 3) meningiomas [1]. The average rate of tumor progression within 5 years after surgical removal ranges between 30 and 60% [2,3,4]. Gross total removal is the most common described predictor of prolonged time to tumor recurrence [5,6]. Furthermore, imaging characteristics, blood-based biomarkers, postoperative radiation therapy, and histopathologic features are highly debated as predictors of progression-free survival [7,8,9,10]. 

Moreover, emerging evidence from single-center studies and meta-analyses have shown that MIB-1 labeling index is an independent predictor of progression-free survival in meningiomas, regardless of the WHO grading of the tumors [11,12,13,14]. However, there are also data showing insignificant results regarding the role of MIB-1 index in recurrence-free survival in Simpson grade 1 resected WHO grade 1 meningiomas. Therefore, the prognostic impact of MIB-1 remains to be further investigated [15]. For preoperative estimation of the MIB-1 labeling index, and hence preoperative estimation of the progression-free survival in newly diagnosed cranial meningiomas, we recently developed a novel scoring system using FibrinOgen, *C*-Reactive protein, Gender, and peritumoral Edema (FORGE score) [16]. Therefore, the scoring sheet includes preoperative systemic inflammatory parameters reflecting the inflammatory burden (i.e., fibrinogen and *C*-reactive protein (CRP)), gender, and the presence of peritumoral edema which are all easily determinable variables and facilitate personalized medical decision making. However, the specific role of the FORGE score in atypical meningiomas and the additional value of the established major histopathological diagnostic criteria regarding tumor recurrence remain unclear so far. WHO grade 2 meningiomas differ significantly from benign WHO grade 1 meningiomas regarding their rate of tumor recurrence. Biomarkers enabling a sufficient scoring system are essential to aid the emerging progress of individualized medicine to generate tailored adjuvant treatment and follow-up interval strategies in the subgroup of atypical meningiomas. 

Against this backdrop, we evaluated the diagnostic performance of the FORGE score in combination with major histopathological diagnostic criteria of atypical meningioma (AT-FORGE score) regarding progression-free survival (PFS). AT-FORGE score was intended to be a proposal for a novel scoring system presenting demographic, inflammatory burden, imaging characteristics and histopathological features in order to facilitate the identification process of a subgroup of atypical meningioma patients with an increased risk of tumor progression.

## 2. Materials and Methods

### 2.1. Study Design and Patient Characteristics

**Figure 1:**
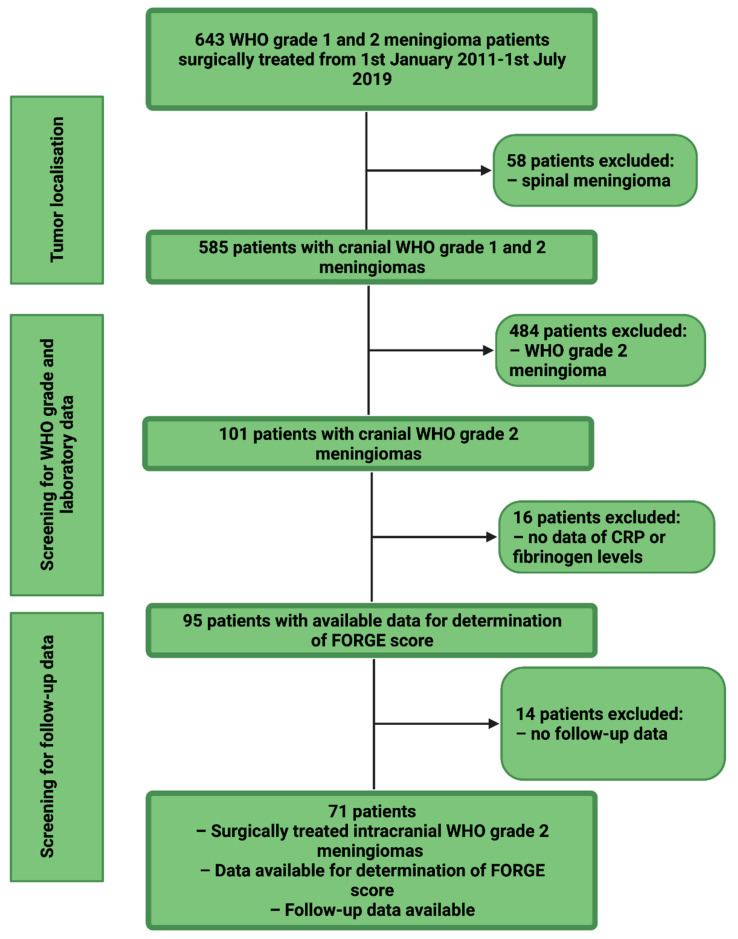
Flow chart displaying the selection process of consecutive atypical meningioma patients between 1 January 2011 and 1 July 2019.

Between January 2011 and July 2019, 643 patients underwent surgery for meningioma (WHO grade 1 or 2) at the department of neurosurgery. Patient data were retrospectively reviewed after institutional review board approval had been obtained. The inclusion criteria of this study were histopathologically confirmed primary diagnosis of an atypical meningioma, intracranial localization, an age greater than 18 years, availability of preoperative systemic inflammatory parameters (fibrinogen and CRP), and surgical therapy via a neurosurgical resection. Patients with a neurofibromatosis type 2-associated meningioma and spinal meningiomas were excluded due to differences regarding histopathological characteristics and proliferative potential [17,18]. Seventy-one patients were included in the final study cohort for the analysis (see Figure 1).

### 2.2. Data Recording

Clinical data including age, sex, comorbidities, Karnofsky performance status (KPS), body mass index (BMI), smoking, acetylsalicylic acid (ASA) intake, tumor size, peritumoral brain edema, tumor growth characteristics, WHO classification based on postoperative neuropathological examination, immunohistochemical examinations, extent of meningioma resection based on the Simpson grading system according to the European Association of Neuro-Oncology (EANO) (Simpson grade 1–3 = gross total resection, Simpson grade 4 = subtotal resection, and Simpson grade 5 = biopsy), and postoperative follow-up data were recorded and saved in a computerized database as previously reported (SPSS, v27 for Mac, IBM Corp., Armonk, NY, USA) [16,19]. Preoperative diagnostic workflow included MR imaging of the brain within 48 h before surgical resection. Tumor size was measured using a diameter-based technique in which the single largest diameter on a single axial preoperative contrast-enhanced T1-weighted MR slice was selected [16,20]. Peritumoral edema was defined as a hyperintense signal adjacent to tumors on T2-weighed MR-images [16,21]. Laboratory parameters were collected using the laboratory information system Lauris (v.17.06.21, Swisslab GmbH, Berlin, Germany). Venous blood samples were routinely taken within 24 h before an operation. These laboratory investigations were performed at constant points in time, which enables a reliable progression-free survival analysis. The routine blood examination before surgery included complete blood count, kidney, and liver tests. The coagulation profile (INR, aPTT) was also examined for every patient. The baseline plasma fibrinogen level was determined by the Clauss method, which involves adding a standard and high concentration of thrombin (Dade^®^ thrombin reagent, Siemens Healthineers, Erlangen, Bavaria, Germany) to platelet poor plasma. Reference curves were used to determine the concentration of fibrinogen. The serum CRP levels were obtained by turbidimetric immunoassays with a CRPL3 reagent (Roche, Basel, Switzerland) [16,22].

### 2.3. Histopathology

Histopathological classification was performed based on the 2016 WHO grading system [1]. All neuropathology reports underwent revisited review to reconfirm that diagnosis was in keeping with these requirements. Immunohistochemical investigations were performed in a similar workflow as described before for paraffin-embedded biopsy tissue specimens [23,24]. The MIB-1 labeling index and cluster of differentiation (CD) 68 immunohistochemical profile was investigated using the following antibodies: Anti-Ki67 (Clone Ki-67P, dilution 1:1000, DAKO, Glostrup, Denmark) and anti-CD68 (Clone KP1, dilution 1:1000, DAKO, Glostrup, Denmark). Semiquantitative investigation of CD68-stainings was performed. Visualization was performed with diaminobenzidine, and neuropathological examination was carried out by a senior neuropathologist (AJB). The MIB-1 index was determined in randomly selected high-power microscopic fields. The numbers of stained and unstained nuclei in the neoplastic cells were determined. The further neuropathological workflows were as previously reported [16,25]. Major diagnostic criteria of atypical meningioma comprise the tumor characteristics “brain invasion” and “number of mitotic figures/10 high power fields” [1].

### 2.4. Follow-Up

Clinical and imaging follow-up regime includes MRI scans at 3 months after surgery as well as on an annual basis for the following years. Earlier clinical and imaging appointments were determined in case of new or progressive neurological deficits as well as radiological characteristics of tumor progression. Recurring atypical meningiomas with radio–clinical interrelationships, regrowing at the local site of the initial surgical resection cavity were considered for analysis. The time to recurrence was defined as the time interval between the initial surgery and the beginning of a subsequent therapy (e.g., radiotherapy or re-do surgery). Radiological regrowing meningiomas without clinical or functional signs, thus not requiring any subsequent treatment options, were not included in the analysis [26].

### 2.5. Statistical Analysis

Data were organized and analyzed using SPSS for Mac (v27.0; IBM Corp, Armonk, NY, USA). Receiver-operating characteristic (ROC) curves were constructed for the FORGE score only and the FORGE score in combination with major histopathological diagnostic criteria of atypical meningiomas in the prediction of meningioma recurrence (AT-FORGE). Cut-off values for the AT-FORGE score were set based on the ROC analysis and the corresponding Youden Index [27]. Normally distributed data are presented as mean with the standard deviation (SD). Preoperative demographic data, comorbidities, tumor features, and laboratory values were compared between the patients with increased AT-FORGE score and normal AT-FORGE score using Fisher’s exact test (two-sided) for categorical data and independent *t*-test for continuous data. Kaplan–Meier charts and log-rank tests of PFS were calculated. Multivariable Cox regression analysis was performed to analyze the PFS.

## 3. Results

### 3.1. Patient Characteristics

Seventy-one patients underwent surgery for cranial atypical meningioma (WHO grade 2) and fulfilled the inclusion criteria. Median age was 63 years (Interquartile range (IQR): 56–75). The cohort consisted of 41 females (57.7%), and 30 males (42.3%; female/male ratio: 1.4:1). The median Karnofsky performance status at preoperative examination was 90 (IQR: 80–100). Further features of atypical meningioma patients are summarized in Table 1.

### 3.2. Tumor Localization, Type of Treatment and Histopathological Characteristics

Location at the convexity (38.0%) was the most common location of atypical meningiomas in the present investigation, followed by falx (25.4%) and the sphenoid wing (14.1%). Multiple meningiomas, sinus invasion, and peritumoral edema were observed in 12 (16.9%), 15 (21.1%), and 46 (64.8%) patients, respectively. Simpson grade I and II resections were performed in 56 (78.9%), whereas 15 (21.1%) underwent Simpson grade III and IV resections. The median MIB-1 labeling index and number of mitotic figures was 5% (IQR: 5–10) and 3 (IQR: 1–6), respectively. Brain invasion was observed in 15 (21.1%) patients. Table 1 summarizes the results.

### 3.3. Value of Major Diagnostic Criteria for Atypical Meningioma on the Prediction of Recurrence

**Figure 2:**
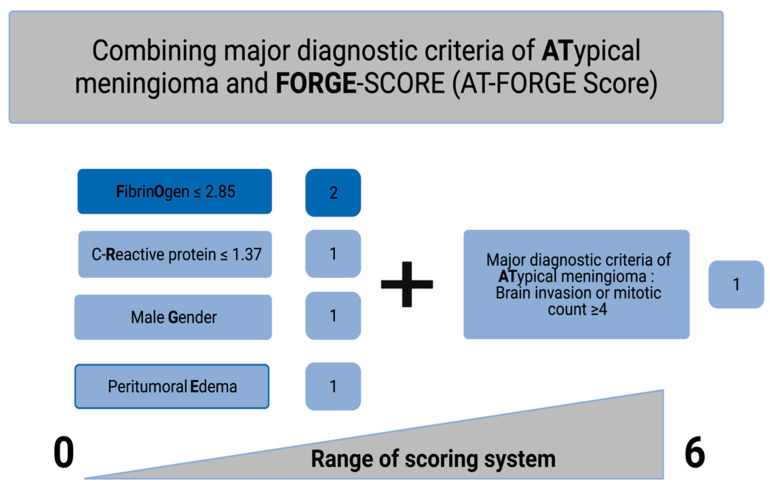
AT-FORGE Score: A simple and quick-to-use add-on to the conventional FORGE score to estimate the risk of tumor progression after surgery for atypical meningioma.

**Figure 3:**
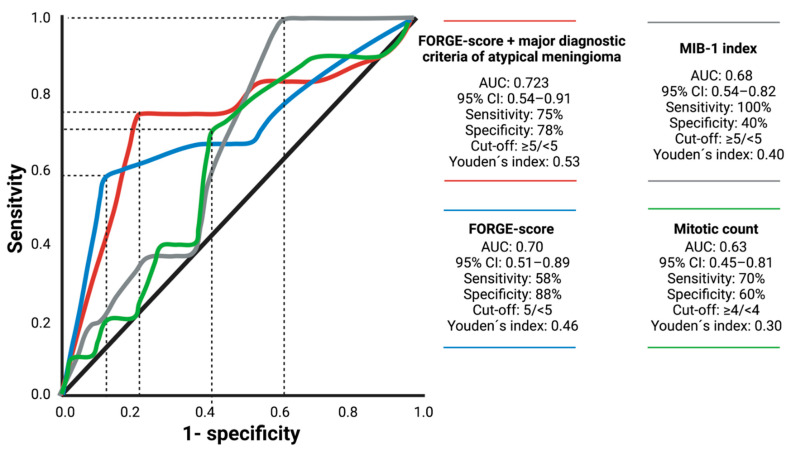
Receiver–operating characteristic curve demonstrating FORGE score (blue line), AT-FORGE score (=FORGE score combined with brain invasion or increased mitotic count; red line), MIB-1 index (grey line), and mitotic count (green line) in the prediction of progression of intracranial atypical meningiomas after surgery. Optimum threshold of the AT-FORGE score in the prediction of tumor progression was found at ≥5 vs. <5. Sensitivity and specificity of AT-FORGE score using optimum cut-off value were 75.0% and 78.0%, respectively. Dashed lines mark the identified optimum cut-off values.

The predictive value of the FORGE score, and the FORGE score combined with the presence of at least one major histopathological diagnostic criteria of atypical meningioma (AT-FORGE) were separately analyzed. Figure 2 displays the corresponding scoring sheet of the AT-FORGE score. The mean MIB-1 labeling index was 6.6 ± 4.1% in the present series. The AUC of the MIB-1 index in the prediction of recurrent atypical meningioma was 0.68 (95% CI: 0.54–0.82, *p* = 0.07). Optimum cut-off value for the MIB-1 index was set at ≥5% (Youden′s index: 0.40; see dashed line marking the optimum cut-off value on the grey line in Figure 3). AUC of the number of mitotic figures predicting recurrence of atypical meningiomas was 0.63 (95% CI: 0.45–0.81, *p* = 0.21). Mean FORGE score was 2.7 ± 1.8. An ROC curve was created, and the area under the ROC curve (AUC) of FORGE score in the prediction of tumor recurrence of atypical meningiomas was determined. The AUC of the FORGE score for recurrent atypical meningioma was 0.70 (95% CI: 0.51–0.89, *p* = 0.03). Sensitivity and specificity of FORGE score for prediction of recurrent atypical meningioma were 58.0% and 88.8%, respectively (Youden’s index: 0.46), with an optimum cut-off value set at 5 vs. <5. Figure 3 shows the ROC curve. The presence of at least one major diagnostic criterion (brain invasion or increased mitotic count (≥4)) yields an additional value of one point to the conventional FORGE score. The AUC of the AT-FORGE score ROC curve for recurrence of atypical meningioma was 0.723 (95% CI: 0.54–0.91, *p* = 0.015). Sensitivity and specificity of AT-FORGE scoring in the prediction of a recurrent atypical meningioma were 75.0% and 78.0%, respectively (Youden’s index: 0.53; see Figure 3). The optimum threshold of the AT-FORGE score in the prediction of tumor progression of atypical meningioma was ≥5 vs. <5.

### 3.4. Comparison of Low vs. High AT-FORGE Score Groups

A total of 49 (69.0%) patients had a AT-FORGE score < 5 and 22 patients (31.0%) had a AT-FORGE score ≥ 5. Age, BMI, preoperative KPS, diabetes, smoking, ASA intake, platelet values, tumor location, presence of multiple meningiomas, presence of sinus invasion, diffuse CD68^+^ macrophage infiltrates, extent of resection, and conduction of adjuvant radiotherapy were homogeneously distributed between both AT-FORGE scoring groups. The baseline patient characteristics and analyses by Fisher´s exact test (two-sided) and independent *t*-test are summarized in Table 2.

### 3.5. AT-FORGE Score in the Prediction of Progression-Free Survival

**Figure 4:**
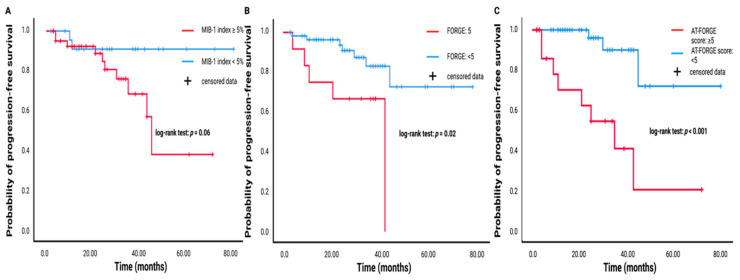
Kaplan–Meier analysis of probability of progression-free survival stratified by MIB-1 index (**A**), FORGE score (**B**), and AT-FORGE score (**C**). Red lines display the arms with shorter time to meningioma progression, whereas the blue lines represent the superior groups regarding progression-free survival. Vertical dashes represent censored data (=progression-free at last clinical visit) within both progression-free survival curves. The time axis is right-censored at 80 months. *p* < 0.001 (log-rank test).

AT-FORGE score combines the FORGE score with major diagnostic histopathological criteria of atypical meningioma. Using an optimum cut-off value of 5, the AT-FORGE score yields a sensitivity of 75.0% and specificity of 78.0% regarding the prediction of a tumor progression of atypical meningioma. The mean time of available follow-up MR-imaging data was 26.0 months (range: 2–80) in the study cohort (*n* = 71). Twelve (12/71; 16.9%) patients with a tumor progression after surgery for atypical meningioma were identified. Patients having a mitotic count ≥ 4 had a mean time to tumor progression of 30.29 months (95% CI: 24.80–35.77), whereas the mean time to progression in patients having a mitotic count < 4 was 68.06 months (95% CI: 57.24–78.88; log-rank test: *p* = 0.09). Mean time to progression of atypical meningioma in patients with a MIB-1 labeling index ≥ 5% (*n* = 45) was 47.64 months (95% CI: 36.46–58.82), whereas patients with a MIB-1 index < 5% (*n* = 26) had a mean time to tumor progression of 73.68 months (95% CI: 65.33–82.03; log-rank test: *p* = 0.06). A FORGE score of 5 points (*n* = 12) resulted in a mean time to tumor progression of 32.42 months (95% CI: 22.68–42.15), whereas patients with a FORGE score < 5 (*n* = 59) had a mean time to atypical meningioma progression of 66.97 months (95% CI: 57.70–76.25; log-rank test: *p* = 0.02). Atypical meningioma patients having a baseline AT-FORGE score of 5 or higher had a mean time to progression of atypical meningioma of 32.76 months (95% CI: 18.80–46.73), and patients with a AT-FORGE score of <5 showed a longer mean time to tumor progression of 71.38 months (95% CI: 61.69–81.07). Log-rank test revealed that patients with a AT-FORGE score ≥ 5 had a significant shorter time to progression of atypical meningioma (*p* < 0.001). Figure 4 displays the Kaplan–Meier curve illustrating the probabilities of progression-free survival for MIB-1 labeling index (<5% vs. ≥5%), FORGE score (<5 vs. 5), and AT-FORGE score (<5 vs. ≥5).

**Figure 5:**
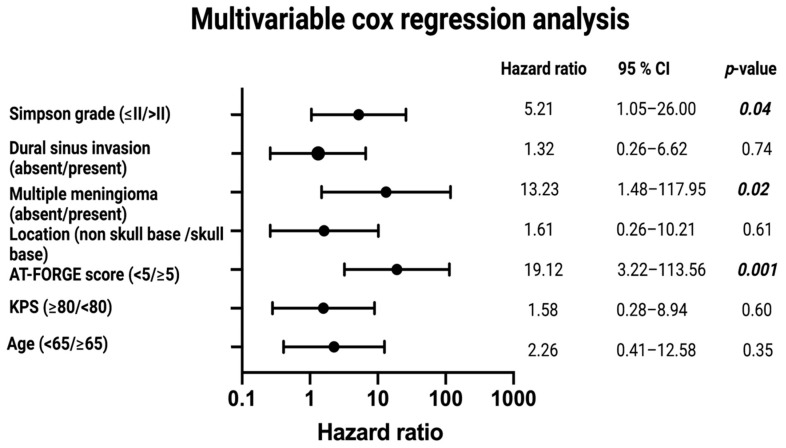
Forest plots from multivariable Cox regression analysis: Simpson grade >II resection, presence of multiple meningiomas, and AT-FORGE score ≥ 5 are independent predictors of progression-free survival. *p*-values in bold and italics display statistically significant results.

We conducted a multivariable cox regression analysis of progression-free survival to determine independent risk factors of patients who underwent surgery for atypical meningioma. Multivariable analysis of PFS was performed with consideration of the following variables (see Figure 5): Age (<65/≥65), Karnofsky performance status (≥80/<80), tumor location (non skull base/skull base), multiple meningiomas (absent/present), dural sinus invasion (absent/present), Simpson grade (≤II/>II), and AT-FORGE score (<5/≥5). The multivariable analysis revealed the variables “Simpson grade > II” (Hazard ratio (HR): 5.2, 95% CI: 1.1–26.0, *p* = 0.04), “multiple meningiomas” (HR: 13.2, 95% CI: 1.5–118.0, *p* = 0.02), and “AT-FORGE score ≥ 5” (HR: 19.1, 95% CI: 3.2–113.6, *p* = 0.001) to be independent predictors for a poor probability of progression-free survival after surgery for atypical meningioma. 

## 4. Discussion

Male sex, younger age at diagnosis, poor Karnofsky performance status, high mitotic count, extent of resection, and involvement of brain nerves are known predictors of shortened time to tumor progression in cranial meningiomas [28]. Recently we have found that the FORGE score can estimate high MIB-1 labeling indices which are strongly associated with tumor progression in meningioma [16,29,30]. However, WHO grade 1 and 2 meningiomas have significantly different potentials to regrow after surgery. Brain invasion and number of mitotic figures are the major diagnostic criteria of atypical meningiomas according to the present WHO grading [1]. Moreover, the presence of three or more of the minor atypical histopathological criteria (increased cellularity, small cells with a high nuclear to cytoplasmic ratio, prominent nucleoli, foci of geographic or spontaneous necrosis, and sheeting) also results in the diagnosis of a WHO grade 2 meningioma. However, the use of minor atypical criteria is highly debated in the literature due to their potential weaker predictive value regarding risk stratification of recurrence compared to major histopathological diagnostic criteria [8]. The present study provides an easily implementable add-on (AT-FORGE score) for the conventional FORGE scoring system in atypical meningioma by additionally considering brain invasion and increased number of mitotic figures. AT-FORGE score seems to be capable of predicting the progression-free survival time in cranial atypical meningiomas.

In the present study, we created ROC curves to investigate the additional value of including the WHO diagnostic criteria “brain invasion” and “mitotic count” in the known FORGE score in atypical meningiomas. The results of the ROC curves and the subsequent analysis of PFS demonstrate that the AT-FORGE score is a sufficient add-on to the conventional FORGE scoring system in atypical meningioma regarding the prediction of PFS. The predictive value of brain invasion in meningioma has been highly debated due to contradictory results regarding overall survival and progression-free survival [31,32,33]. Nevertheless, evidence of mitotic count as the second major diagnostic criteria in the prediction of the clinical endpoint “local progression-free survival” is stronger compared to brain invasion [34,35].

Multivariable cox regression analysis identified Simpson grade > II resection, presence of multiple meningiomas, and a AT-FORGE score ≥ 5 as independently and significantly associated with shortened time to tumor progression of atypical meningioma. Residual tumor tissue is known to be highly influential on recurrence of atypical meningioma [5,36,37]. Hence, the treatment of choice for atypical meningioma should be a complete resection (Simpson grade ≤ II), whenever possible with preservation of the neurological functioning. Furthermore, the presence of multiple meningiomas (≥2 cranial meningioma) was also significantly and independently associated with local tumor progression of atypical meningioma. Several retrospective studies reported that the prognosis of patients with multiple meningiomas does not differ from that of solitary meningiomas, except in the case of radiation-induced multiple meningiomas and neurofibromatosis type 2—associated meningiomas in children and adolescents [38,39]. However, a recent population-based survival analysis of 99,918 cases from 1975 to 2017 revealed that an increasing number of meningiomas has a significant negative influence on overall survival [40]. Additionally, they found that female patients with multiple meningiomas had an increased overall survival and a reduced risk to develop multiple meningiomas.

AT-FORGE score displays the combination of inflammatory burden (plasma fibrinogen and serum CRP), male sex, and peritumoral brain edema with the major diagnostic criteria of atypical meningioma. Plasma fibrinogen and serum CRP are both linked to the interleukin-6 (IL-6) gene promoter [41]. It has been found that human meningioma cells are capable to secrete IL-6 which can act as an autocrine inhibitory regulator of the growth of neoplastic cells [42]. Additionally, the administration of anti-IL-6 antibodies resulted in an enhancement of the growth of meningiomas. IL-6 might also have a direct influence on the integrity of the blood-brain barrier of intracranial arteries and might result in a change of the structure and the permeability of the endothelium [43,44]. Those pathophysiological pathways enable the secretion of CRP in hepatocytes by induction of IL-6 secreted by meningiomas [45]. CRP is capable to polarize human macrophages to an M1 macrophage and simultaneously inhibits the transformation to the M2 phenotype which were found to be pro-tumor macrophages enhancing tumor growth and recurrence in meningiomas [46,47]. However, there are also contradictory data which showed that patients with increased preoperative fibrinogen levels had a shorter time to tumor progression of atypical meningiomas [10]. IL-6 as a stimulator of CRP and fibrinogen secretion can act multifunctionally by the mediation of inflammation or induction of cellular differentiation [48,49]. The function of IL-6 in meningioma seems to be ambiguously as it might enhance tumor growth in approximately 60% of tumors, whereas other investigations found an inhibitory role on proliferative activity of neoplastic cells [50,51]. Male sex has been identified in several studies as a risk factor of tumor recurrence [52,53] A recent study by Escribano et al. [53] retrospectively investigated 125 patients with parasagittal meningiomas and identified male sex as an independent risk factor of recurrence in a binary logistic regression model. The role and development of peritumoral brain edema are still highly discussed in the literature. Peritumoral brain edema has been found as a predictor of recurrence in cranial meningiomas [54,55]. Additionally, peritumoral brain edema is significantly associated with a higher MIB-1 labeling index illustrating the proliferative activity of meningiomas [56]. 

The newly created proposal of the AT-FORGE score as an add-on to the conventional FORGE score in atypical meningioma provides a novel scoring system to estimate the time to tumor progression after surgery for intracranial atypical meningiomas. To date, follow-up imaging after subtotal resection of meningiomas is still the standard treatment in most institutions [57] despite several studies revealed strong evidence for an adjuvant radiotherapy after both subtotal or gross total resection of atypical meningioma [58,59,60]. However, the optimal postoperative treatment strategy for atypical meningiomas has not been exactly identified yet. Against this backdrop, the present score might be of paramount importance to identify individuals with an atypical meningioma who are at increased risk of tumor recurrence and potential benefit from an adjuvant radiotherapy despite the risks of radiation-induced toxicity [61,62]. EANO-guidelines recommend MR follow-up images every 6 months for 5 years after surgery and afterwards on an annual base [63]. The AT-FORGE score might be a useful tool for a comprehensive consultation with patients and their relatives to determine more stringent follow-up intervals if an increased risk for a tumor recurrence was calculated by using the AT-FORGE score.

The present investigation has several limitations. Data were acquired retrospectively from a highly selective and homogeneous collective. However, the results of the present investigation have to be interpreted with caution due to a single-center experience. A multicenter prospective trial with a stringent design should provide an external validation of the FORGE score and its add-on AT-FORGE score in cranial atypical meningiomas.

## 5. Conclusions

The present investigation demonstrated a strong association between the AT-FORGE score and shortened time to tumor progression in intracranial atypical meningioma patients. The AT-FORGE score might provide a useful tool for determination of postoperative follow-up imaging intervals, and risk–benefit assessment in the adjuvant therapy options for atypical meningioma patients.

## Figures and Tables

**Table 1:** Patient characteristics (*n* = 71).

Median Age (IQR) (in y)	63 (56–75)
Sex Female Male	41 (57.7%) 30 (42.3%)
Median preoperative KPS (IQR)	90 (80–100)
Tumor location	
Convexity	27 (38.0%)
Falx	18 (25.4%)
Sphenoid wing	10 (14.1%)
Posterior fossa	8 (11.3%)
Frontobasal	7 (9.9%)
Others	1 (1.4%)
Multiple meningiomas	12 (16.9%)
Sinus invasion	15 (21.1%)
Peritumoral edema	46 (64.8%)
Simpson grade Simpson grade I&II Simpson grade ≥ III	56 (78.9%) 15 (21.1%)
Brain invasion	15 (21.1%)
High mitotic count (≥4)	35 (49.3%)
Brain invasion and/or high mitotic count	50 (70.4%)
Minor atypical criteria only	21 (29.6%)
Median MIB-1 (IQR)	5 (5–10)
Median mitotic count (IQR)	3 (1–6)
Adjuvant radiotherapy	3 (4.2%)

**Table 2:** Univariable analysis of demographic, clinical, laboratory, imaging, and histopathological features between patients with low and high AT-FORGE score.

Variable	AT-FORGE Score: <5 (*n* = 49)	AT-FORGE Score: ≥5 (*n* = 22)	*p*-Value
Age (mean ± SD)	62.5 ± 13.1	67.1 ± 14.3	0.19
BMI (mean ± SD)	27.2 ± 5.7	26.5 ± 2.7	0.49
Preoperative KPS (mean ± SD)	88.4 ± 12.8	82.7 ± 12.8	0.09
Diabetes (yes/no)	9/40	1/21	0.16
Smoking (yes/no)	14/32	8/14	0.99
ASA intake (yes/no)	9/40	6/16	0.53
Platelet count (mean ± SD)	252.5 ± 64.1	224.9 ± 49.69	0.08
MPV (mean ± SD)	10.9 ± 1.2	10.5 ± 0.7	0.15
Location (Skull base/Non skull base)	17/32	6/16	0.59
Sinus invasion (present/absent)	8/41	7/15	0.21
Multiple meningiomas (present/absent)	8/41	4/18	0.99
Diffuse CD68^+^ macrophage infiltrates (available in 60 patients)	27/15	9/9	0.39
Simpson grade (≤II/>II)	41/8	15/7	0.21
Adjuvant radiotherapy	1/48	2/20	0.23

## Data Availability

All data are included in this manuscript.
